# Association of triglyceride glucose-body mass index with Alzheimer’s disease pathology, cognition and brain structure in non-demented people

**DOI:** 10.1038/s41598-024-67052-3

**Published:** 2024-07-12

**Authors:** Zihao Zhang, Xin Chen, Zehu Sheng

**Affiliations:** 1 Medical College, Qingdao University, Qingdao, 266000 China; 2 School of Athletic Performance, Shanghai University of Sport, Shanghai, 200438 China; 3 Chongming District Sports School, Shanghai, 202150 China; 4 Department of Geriatrics, The First Affiliated Hospital of Chongqing Medical University, Chongqing Medical University, Chongqing, 400016 China

**Keywords:** Alzheimer’s disease, Triglyceride glucose-body mass index, Pathology, Cognition, Brain structure, Insulin resistance, Cognitive ageing, Neurogenesis

## Abstract

The relationship between the triglyceride glucose-body mass index (TyG-BMI) index and Alzheimer’s disease (AD) pathology, cognition, and brain structure remains unclear. This study aimed to investigate these associations, focusing on cerebrospinal fluid (CSF) biomarkers, cognitive measures, and brain imaging data. Eight hundred and fifty-five non-demented participants were included. Linear regression was used to explore associations between the TyG-BMI index and AD pathology, cognition, and brain structure. The association between the TyG-BMI index and AD risk was assessed using Kaplan–Meier and Cox proportional hazards models. Longitudinal relationships were assessed using linear mixed-effects models. Mediation analyses were conducted to examine AD pathology’s potential mediating role between the TyG-BMI index and cognition as well as brain structure. In the linear regression analyses, higher TyG-BMI levels were associated with increased Aβ_42_ and decreased Tau, pTau, Tau/Aβ_42_, pTau/Aβ_42_, and pTau/Tau. Positive correlations were observed with mini-mental state examination (MMSE), memory (MEM), executive function (EF), and the volumes of the hippocampus, entorhinal cortex, and middle temporal regions, while negative correlations were found with Alzheimer’s Disease Assessment Scale (ADAS). Longitudinally, the TyG-BMI index was inversely associated with ADAS, and positively with MMSE, MEM, EF, hippocampus, entorhinal, and middle temporal. High TyG-BMI levels were correlated with lower AD risk (HR 0.996 [0.994, 0.999]). Mediation analyses revealed AD pathology mediated the association between TyG-BMI index and cognition as well as brain structure. Additionally, the TyG-BMI index could mediate cognitive changes by influencing brain structure. The TyG-BMI index is associated with AD pathology, cognition, and brain structure.

## Introduction

Alzheimer’s disease (AD) is a prevalent neurodegenerative disorder profoundly impacting cognition and functionality in affected individuals^1^. With the aging population, AD poses an increasingly significant burden on individuals, families, and society. Research underscores the critical role of metabolic disturbances, such as insulin resistance (IR) and obesity, in the pathogenesis of AD^2,3^. Insulin resistance, characterized by impaired cellular response to insulin, has emerged as a pivotal risk factor for AD development, disrupting normal neuronal function and precipitating neuroinflammation, amyloid plaque accumulation, and neurofibrillary tangle formation^4–6^.

Traditional insulin sensitivity assays, such as the insulin tolerance test, are commonly used to assess insulin sensitivity^7^. However, these methods require stringent laboratory conditions and prolonged monitoring. The triglyceride glucose (TyG) index offers a simpler and more cost-effective alternative for evaluating IR^8^. Calculated from fasting blood glucose (FBG) and triglyceride (TG), this index provides a comprehensive reflection of metabolic health^9^. Prior studies have highlighted an association between the TyG index and AD risk, but establishing a causal relationship remains challenging in observational studies^10–12^. Furthermore, existing research lacks systematic evidence examining all three aspects of AD pathology, cognitive measures, and brain structure.

To better understand the relationship between metabolic dysfunction and AD, we employed the composite indicator triglyceride glucose-body mass index (TyG-BMI) index, which combines the TyG index with BMI, enhancing its predictive power for IR and metabolic syndrome^13^. By incorporating BMI, which reflects obesity, the TyG-BMI index captures both systemic metabolic disturbances and IR associated with obesity^14^. Previous studies have shown that the TyG-BMI index has a good concordance with IR assessments such as homeostasis model assessment of insulin resistance, and the area under the curve for evaluating IR is greater with TyG-BMI compared to TyG alone^14,15^.

However, there is a lack of studies on the association between TyG-BMI index and AD. Our main objective was to examine the associations between the TyG-BMI index and AD pathology, cognition, and brain structure. We hypothesized that TyG-BMI levels would be associated with specific AD pathological biomarkers, such as Aβ_42_, Tau, and pTau. These pathological changes could potentially affect cognition, including memory and executive function, and result in volume changes in brain regions such as the hippocampus, entorhinal cortex, and middle temporal regions. Additionally, we sought to investigate whether the TyG-BMI index influences cognition and brain structure through its effects on AD pathology.

## Methods

### Study sample

A total of 855 non-dementia adults were gathered from the Alzheimer’s Disease Neuroimaging Initiative (ADNI) database. Patients without available BMI, TG, FBG, blood pressure, history of diabetes, history of cardiovascular disease (CVD), AD pathology, global cognition and cognitive domain measures, and dementia were excluded. These participants, ranging in age from 54 to 91 years, provided us with comprehensive data from the ADNI study, including essential clinical characteristics, biochemical biomarkers of AD, imaging data, and cognitive assessment data. More information is available at http://adni.loni.usc.edu.

### Measurements of laboratory parameters

The ADNI database provided all laboratory and anthropometric parameters and medical history data. FBG and TG levels were measured. *APOEε4* genotyping was conducted at the ADNI Biomarker Core Laboratory, University of Pennsylvania, to identify participants with at least one *APOEε4* allele, designating them the *APOEε4* positive status^16^. Height and weight were measured at baseline for all participants. BMI was computed utilizing the equation: body weight (kilograms) divided by height (meters)^2^. The TyG-BMI index was calculated applying the following formula: ln [FBG (mg/dL) × TG (mg/dL)/2] × BMI^14^. The included population lacked a diagnosis of diabetes mellitus and hypertension, and we used FBG ≥ 126 mg/dL as the basis for diabetes mellitus^17^, systolic blood pressure ≥ 140 mmHg or diastolic blood pressure ≥ 90 mmHg as the basis for the diagnosis of hypertension^18^.

### Definition of incident AD and cognitive measures

AD diagnosis was confirmed in individuals meeting the established criteria for probable AD set by the US National Institute of Neurological and Communicative Disorders and Stroke-Alzheimer’s Disease and Related Disorders Association criteria^19^. The ADNI utilized various scales to evaluate cognitive abilities, encompassing global cognition assessed through the Mini-Mental State Examination (MMSE) and Alzheimer’s Disease Assessment Scale (ADAS), along with specific cognitive domains like executive function (EF) and memory function (MEM)^20^.

### Measurements of AD biomarkers

Cerebrospinal fluid (CSF) specimens were obtained via lumbar puncture and promptly transferred to 10-mL polypropylene tubes. Within a two-hour window, these samples underwent transportation to the laboratory. Subsequently, the CSF samples underwent centrifugation at 2000 *g* for 10 min. Samples not detected in time for thawing and freezing procedures did not exceed two cycles prior to analysis^16^. The INNO-BIA AlzBio3 immunoassay (Innogenetics-Fujirebio, Ghent, Belgium) was employed to quantify CSF concentrations of Aβ_42_, Tau, and pTau (pg/mL). We used Tau/Aβ_42_ and pTau/Aβ_42_ because they are better predictors of brain Aβ_42_ deposition and cognitive decline than tau and pTau expressed alone^21,22^.

### MRI assessment

The protocol of the ADNI FreeSurfer-based pipeline has been detailed in previous publications^23^. Initial preprocessing of the MRI T1-weighted image involved intensity normalization and gradient expansion. Following this, non-brain tissue removal was performed using a hybrid watershed/surface deformation approach. Automatic Talairach transform was then employed to segment subcortical white matter and deep gray matter structures. We selected the hippocampus, entorhinal cortex, and middle temporal regions for our analysis due to their critical involvement in AD pathology, as they are among the first regions to show signs of atrophy. These regions are crucial for memory formation and have been extensively studied in AD research. Hippocampus, entorhinal and middle temporal volumes were extracted for this study, and 744, 740, 740 participants were included in the analysis of TyG-BMI index with brain imaging, respectively.

### Statistical analyses

For continuous variables, mean (standard deviation) or median (interquartile range) were used to represent normal or non-normal distributions, respectively. Analysis of Variance or the Kruskal–Wallis test was employed for analysis accordingly. Categorical variables were depicted as numbers (n) and percentages (%), with chi-square tests employed for assessment. Outliers exceeding three standard deviations were eliminated from the statistical analysis involving the TyG-BMI index.

Initially, relationships between the TyG-BMI index (independent variable) and AD pathology, cognition, and brain structure (dependent variable) were explored via multivariable linear regression models. Analyses were conducted both with and without adjustment for covariates to assess the robustness of the findings. To identify potential interaction effects, we examined interaction terms between TyG-BMI and various covariates. For covariates where the interaction term was significant (*P* < 0.05) or indicated a potential interaction (*P* < 0.1), we conducted stratified analyses to further explore the associations within these subgroups.

The TyG-BMI index was also categorized into tertiles (low [T1], medium [T2], high [T3]). Using multivariable logistic regression (MLR), we compared the associations of the second and third tertiles with the first tertile (reference) in relation to AD pathology, cognition, and brain structure. Additionally, to explore the connection between the TyG-BMI index and the likelihood of AD development, we calculated the cumulative incidence rate through the Kaplan–Meier method and estimated the hazard ratio (HR) with a 95% confidence interval (CI) for AD using the Cox proportional hazards model. Both continuous TyG-BMI values and tertile groups were analyzed to assess their impact on AD risk.

We employed linear mixed-effects models to delineate the longitudinal relationships between baseline TyG-BMI index and longitudinal measures of AD pathology, cognition, and brain structure. Interaction terms between TyG-BMI index and time were included to assess changes over time.

For mediation analyses, we followed Baron and Kenny’s approach to ascertain whether AD pathology mediated the association of the TyG-BMI index with cognition and brain structure, and whether the TyG-BMI index partially affected cognition through brain structure^24^. We established a mediating effect if the following conditions were met: (1) the independent variable (IV) correlated with mediator variable (MV); (2) the TyG index correlated with dependent variable (DV); (3) MV correlated with DV; and (4) the correlation between the IV and cognition was attenuated after introducing MV as mediators into the regression model. In addition, the magnitude of attenuation or indirect effects was estimated and significance was ascertained via 10,000 self-directed iterations.

In all analyses, adjustments were made for age, sex, ethnicity, education, *APOEε4* carrier status, cognitive diagnosis, smoking, drinking, hypertension, and CVD as covariates. Additionally, intracranial volume was included as a covariate in MRI measurements analyses. Given that all outcome variables were standardized to z-scores in the model, the coefficient represents the standardized effect. Statistical analyses were performed using R version 4.2.0, with statistical significance set at *P* < 0.05 for all analyses.

### Ethical approval

The entire approval for this study was obtained from the Eisai Ethics Committee (2017-0433). Following the Declaration of Helsinki, written informed consent was obtained from all participants or their guardians.

## Results

### Participants’ characteristics

Eight hundred and fifty-five non-demented participants were included, with a mean age of 73.03 ± 7.13 years, 44.0% female, and a maximum follow-up of 16 years. Individuals displaying elevated TyG-BMI index levels exhibited several distinct demographic and clinical characteristics. They tended to be younger, had lower levels of education, were less likely to be of whites, and were less likely to carry the *APOEε4* gene. Additionally, they showed a higher prevalence of CVD, diabetes, and hypertension. Significant difference in Aβ_42_, Tau, pTau, Tau/Aβ_42_, pTau/Aβ_42_, pTau/Tau, ADAS hippocampus, entorhinal between groups. The T2, T3 group had higher CSF Aβ_42_ and lower Tau and pTau compared to the T1 group. For brain structure, the hippocampus, entorhinal, and middle temporal were larger in the group with high TyG-BMI index levels (Table 1).

**Table 1:** Characteristics based on the TyG-BMI index tertiles of 855 participants.

Characteristic	Overall	Tertile1	Tertile2	Tertile3	*P*
Number	855	285	285	285	
Age	73.03 (7.13)	73.66 (7.17)	73.64 (7.09)	71.79 (6.99)	0.001
Education	16.15 (2.71)	16.64 (2.58)	16.15 (2.73)	15.67 (2.76)	< 0.001
Sex (%)					0.080
Female	376 (44.0)	140 (49.1)	114 (40.0)	122 (42.8)	
Male	479 (56.0)	145 (50.9)	171 (60.0)	163 (57.2)	
Ethnicity (%)					0.046
Other	54 (6.3)	16 ( 5.6)	12 ( 4.2)	26 ( 9.1)	
White	801 (93.7)	269 (94.4)	273 (95.8)	259 (90.9)	
APOE ε4 (%)					0.006
APOE ε4 (−)	495 (57.9)	150 (52.6)	159 (55.8)	186 (65.3)	
APOE ε4 (+)	360 (42.1)	135 (47.4)	126 (44.2)	99 (34.7)	
Dignosis (%)					0.900
CN	257 (30.1)	88 (30.9)	83 (29.1)	86 (30.2)	
MCI	598 (69.9)	197 (69.1)	202 (70.9)	199 (69.8)	
CVD (%)					< 0.001
No	289 (33.8)	123 (43.2)	93 (32.6)	73 (25.6)	
Yes	566 (66.2)	162 (56.8)	192 (67.4)	212 (74.4)	
Diabetes (%)					< 0.001
No	749 (87.6)	266 (93.3)	251 (88.1)	232 (81.4)	
Yes	106 (12.4)	19 ( 6.7)	34 (11.9)	53 (18.6)	
Hypertension (%)					0.142
No	506 (59.2)	182 (63.9)	163 (57.2)	161 (56.5)	
Yes	349 (40.8)	103 (36.1)	122 (42.8)	124 (43.5)	
Drinking (%)					0.917
No	819 (95.8)	274 (96.1)	272 (95.4)	273 (95.8)	
Yes	36 ( 4.2)	11 ( 3.9)	13 ( 4.6)	12 ( 4.2)	
Smoking (%)					0.428
No	524 (61.3)	180 (63.2)	166 (58.2)	178 (62.5)	
Yes	331 (38.7)	105 (36.8)	119 (41.8)	107 (37.5)	
Glucose (mg/dL)	100.93 (23.64)	95.78 (14.72)	99.24 (19.11)	107.78 (31.96)	< 0.001
Triglycerides (mg/dL)	146.01 (107.91)	97.45 (42.74)	138.28 (66.00)	202.30 (152.40)	< 0.001
TyG index	8.73 (0.59)	8.35 (0.43)	8.72 (0.47)	9.12 (0.58)	< 0.001
BMI (kg/m2)	28.27 (4.42)	24.14 (1.97)	27.73 (1.74)	32.94 (3.53)	< 0.001
TyG-BMI	247.62 (46.27)	201.52 (17.37)	241.26 (10.01)	300.08 (33.17)	< 0.001
AD pathology					
Aβ42 (pg/mL)	1122.10 (606.37)	1010.84 (582.50)	1119.42 (608.79)	1236.03 (608.50)	< 0.001
Tau (pg/mL)	270.07 (120.10)	282.23 (124.84)	279.46 (123.92)	248.53 (108.31)	0.001
pTau (pg/mL)	25.80 (13.45)	27.35 (13.77)	26.79 (14.15)	23.28 (12.01)	< 0.001
Tau/Aβ42	0.33 (0.26)	0.38 (0.26)	0.34 (0.27)	0.27 (0.23)	< 0.001
pTau/Aβ42	0.03 (0.03)	0.04 (0.03)	0.03 (0.03)	0.03 (0.02)	< 0.001
pTau/Tau	0.09 (0.01)	0.09 (0.01)	0.09 (0.01)	0.09 (0.01)	< 0.001
Cognition					
MMSE (IQR)	29.00 (3.00)	29.00 (3.00)	29.00 (3.00)	29.00 (3.00)	0.059
ADAS (IQR)	8.00 (5.67)	9.00 (5.33)	8.33 (5.33)	7.00 (6.00)	0.017
MEM (IQR)	0.46 (1.08)	0.43 (1.20)	0.43 (1.01)	0.51 (1.01)	0.034
EF (IQR)	0.35 (1.26)	0.31 (1.33)	0.35 (1.21)	0.43 (1.18)	0.608
Brain structure					
Hippocampus (mm3)	7037.83 (1098.63)	6907.03 (1041.04)	6958.67 (1150.73)	7251.97 (1074.47)	0.001
Entorhinal (mm3)	3646.17 (707.68)	3550.51 (714.05)	3633.67 (711.48)	3759.56 (683.49)	0.005
Middle Temporal (mm3)	20,076.19 (2774.66)	19,762.92 (2717.40)	20,228.43 (2878.79)	20,242.11 (2703.95)	0.091
Intracranial volume (mm3)	1,527,091.82 (164,907.44)	1,515,485.34 (155,151.91)	1,542,361.67 (175,565.06)	1,523,482.40 (162,826.57)	0.138

Data are mean (SD), n (%), or median (IQR).

TyG-BMI index, triglyceride glucose-body mass index; AD, Alzheimer’s Disease; MMSE, mini-mental state examination, ADAS, Alzheimer’s Disease Assessment Scale-Cognitive Subscale Question; EF, executive function; MEM, memory function. Aβ42, Amyloid-42; pTau, phosphorylated-tau; Tau, total-tau; APOE ε4, Apolipoprotein E; CN, cognitive normal; MCI, mild cognitive impartment; CVD, cardiovascular disease.

### Association of TyG-BMI index with AD biomarkers, cognitive measures and brain structure

**Figure 1:**
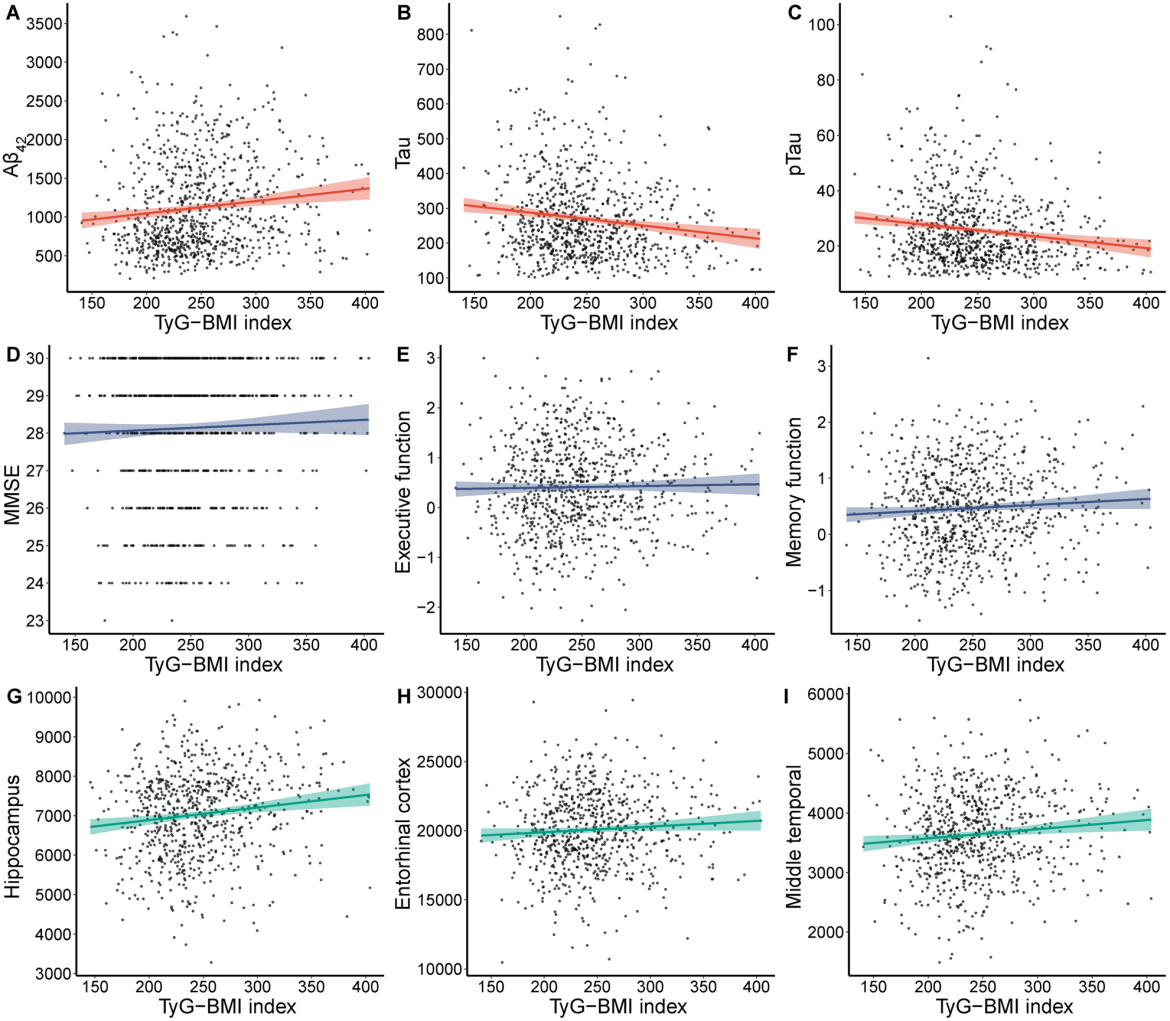
Associations of TyG-BMI index and Alzheimer’s disease biomarkers, cognition and brain structure. TyG-BMI index, triglyceride glucose-body mass index; Aβ_42_, Amyloid-42; pTau, phosphorylated-tau; Tau, total-tau; MMSE, mini-mental state examination.

An increased TyG-BMI index showed a significant association with higher levels of Aβ_42_ (β = 0.096, *P* = 0.003). Conversely, as the TyG-BMI index increased, Tau (*β* = − *0.134, P* = *6.38E-5*), pTau (*β* = − *0.140, P* = *2.62E-5*), Tau/Aβ_42_ (*β* = − *0.145, P* = *3.84E−6*), pTau/Aβ_42_ (*β* = − *0.144, P* = *5.38E−6*), and pTau/Tau (*β* = − *0.127, P* = *1.42E−4*) gradually decreased (Fig. 1A–C, Supplementary file Table 1). The TyG-BMI index exhibited positive correlations with MMSE (*β* = *0.071, P* = *0.030*), MEM (*β* = *0.095, P* = *0.001*), EF (*β* = *0.068, P* = *0.042*), hippocampus (*β* = *0.129, P* = *3.17E−4*), entorhinal (*β* = *0.098, P* = *0.006*), and middle temporal (*β* = *0.077, P* = *0.022*), and negative correlations with ADAS (*β* = − *0.093, P* = *0.002*) (Fig. 1D–I, Supplementary file Table 1). To further understand the association between different levels of TyG-BMI index and AD biomarkers, cognition, and brain structure, we categorized the TyG-BMI index into tertiles and analyzed them using multivariate logistic regression, the results of which are presented in Table 2.

**Table 2:** Association of TyG-BMI index and pathology, cognition as well as brain structure.

Characteristic	Tertile1		Tertile2		Tertile3	
	OR (95%CI)	*P*	OR (95%CI)	*P*	OR (95%CI)	*P*
AD pathology						
Aβ_42_	Reference		1.217 (1.049, 1.413)	0.001	1.353 (1.161, 1.576)	1.19E−04
Tau	Reference		0.971 (0.831, 1.135)	0.711	0.785 (0.669, 0.922)	0.003
pTau	Reference		0.950 (0.815, 1.109)	0.519	0.767 (0.654, 0.899)	0.001
Tau/Aβ_42_	Reference		0.871 (0.754, 1.006)	0.061	0.731 (0.614, 0.827)	9.06E−06
pTau/Aβ_42_	Reference		0.878 (0.759, 1.106)	0.081	0.717 (0.617, 0.833)	1.56E−05
pTau/Tau	Reference		0.847 (0.726, 0.988)	0.035	0.727 (0.620, 0.852)	9.32E−05
Cognition						
MMSE	Reference		0.951 (0.817, 1.107)	0.516	1.157 (0.990, 1.353)	0.067
ADAS	Reference		0.865 (0.745, 1.004)	0.057	0.751 (0.645, 0.876)	2.75E−05
MEM	Reference		1.118 (0.976, 1.282)	0.109	1.300 (1.129, 1.496)	2.78E−04
EF	Reference		1.167 (1.000, 1.362)	0.051	1.238 (1.055, 1.452)	0.009
Brain structure						
Hippocampus	Reference		1.064 (0.903, 1.255)	0.457	1.360 (1.148, 1.612)	4.10E−04
Entorhinal	Reference		1.084 (0.920, 1.276)	0.335	1.342 (1.131, 1.592)	7.76E−04
MidTemp	Reference		1.130 (0.970, 1.318)	0.117	1.227 (1.045, 1.439)	0.013

TyG-BMI index, triglyceride glucose-body mass index; AD, Alzheimer’s Disease; MMSE, mini-mental state examination, ADAS, Alzheimer’s Disease Assessment Scale-Cognitive Subscale Question; EF, executive function; MEM, memory function. Aβ42, Amyloid-42; pTau, phosphorylated-tau; Tau, total-tau.

All factors adjusted for sex, age, ethnicity, Apolipoprotein E4, cognitive dignosis, diabetes, education, smoking, drinking, hypertension and cardiovascular disease, brain structure additionally adjusted for intracerebral volume.

In the interaction analysis, we found significant interactions between age and TyG-BMI index with Aβ_42_, cognitive diagnosis and TyG-BMI index with Tau, and MMSE and TyG-BMI index. In the subgroup analyses, we observed significant associations between TyG-BMI index and AD pathologies among specific participant subgroups (Supplementary file Table 2). Specifically, we found that in individuals over 60 years old, males, those with mild cognitive impairment, and those not carrying the *APOEε4* gene, the associations between TyG-BMI and AD biomarkers were consistent with those observed in the overall population (Supplementary file Table 3). Subgroup analyses examining cognition and brain structure produced findings consistent with those of AD biomarkers. However, the association was notably more significant among participants lacking the *APOEε4* gene, and no significant differences were observed by gender (Supplementary file Tables 4 and 5).

### Longitudinal relationship between TyG-BMI index with cognitive measures and brain structure

**Figure 2:**
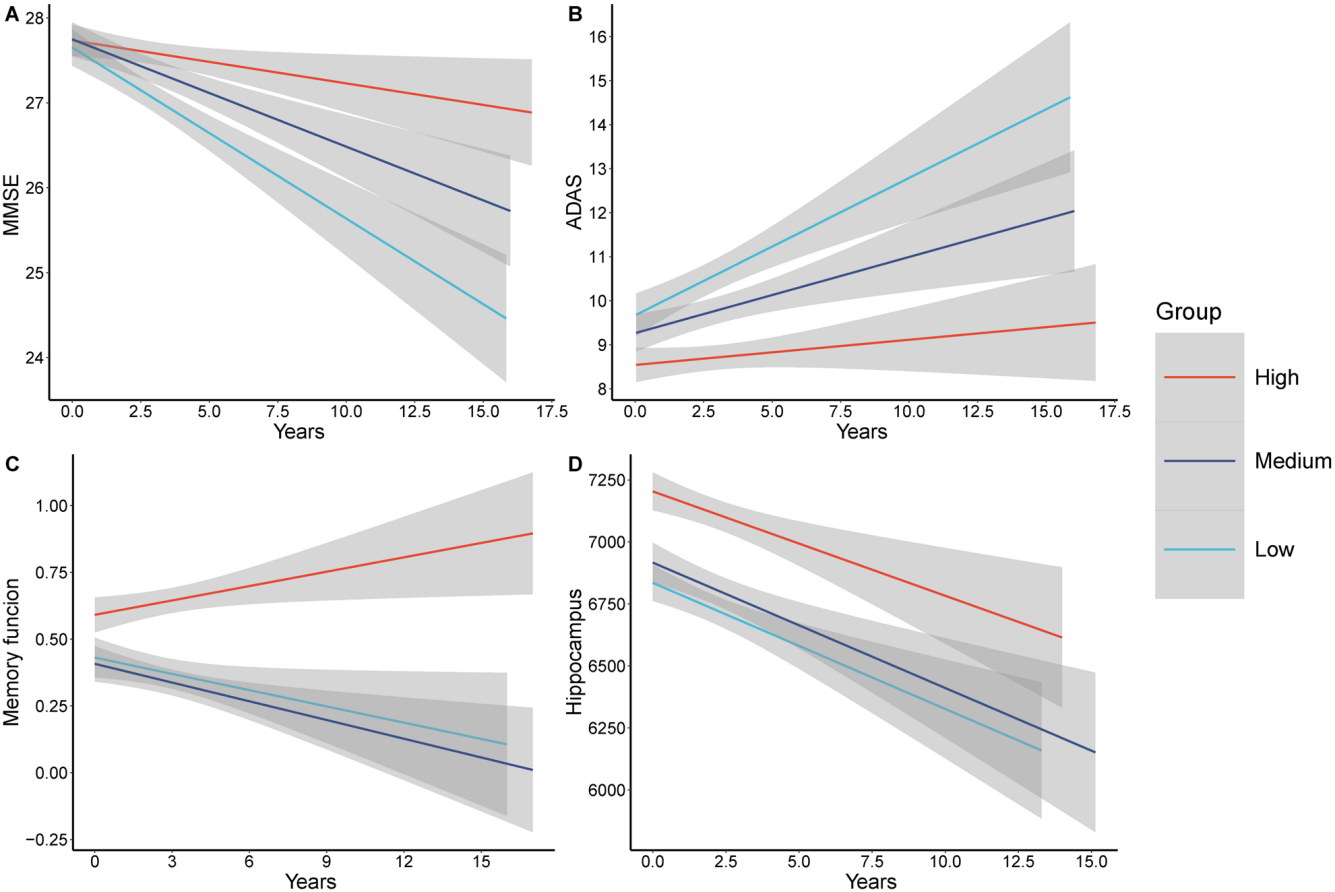
Longitudinal relationship between different TyG-BMI index levels with cognitive measures and brain structure. MMSE, mini-mental state examination, ADAS, Alzheimer’s Disease Assessment Scale-Cognitive Subscale Question.

Longitudinally, we found associations between the TyG-BMI index and MMSE (*β* = *0.045, P* = *6.36E-5*), ADAS (*β* = − *0.046, P* = *5.88E−6*), EF (*β* = *0.015, P* = *0.011*), MEM (*β* = *0.024, P* = *3.36E−5*), entorhinal (*β* = *0.014, P* = *0.007*) and middle temporal volume (*β* = *0.013, P* = *0.036*) (Supplementary file Table 6). MMSE scores exhibited a slower decline over time in the medium and high groups compared to the low group. Similarly, ADAS scores showed a slower rate of increase in the medium and high groups. Regarding MEM, there was a gradual decline in the low and medium groups, while the high group demonstrated a gradual improvement. Furthermore, hippocampal volume exhibited a slower rate of decline in the medium and high groups (Fig. 2). The number of AD pathology, cognition, and imaging data included in the analysis during the follow-up time is shown in Supplementary file Table 7.

### Association of TyG-BMI index with survival rates and risk of AD

**Figure 3:**
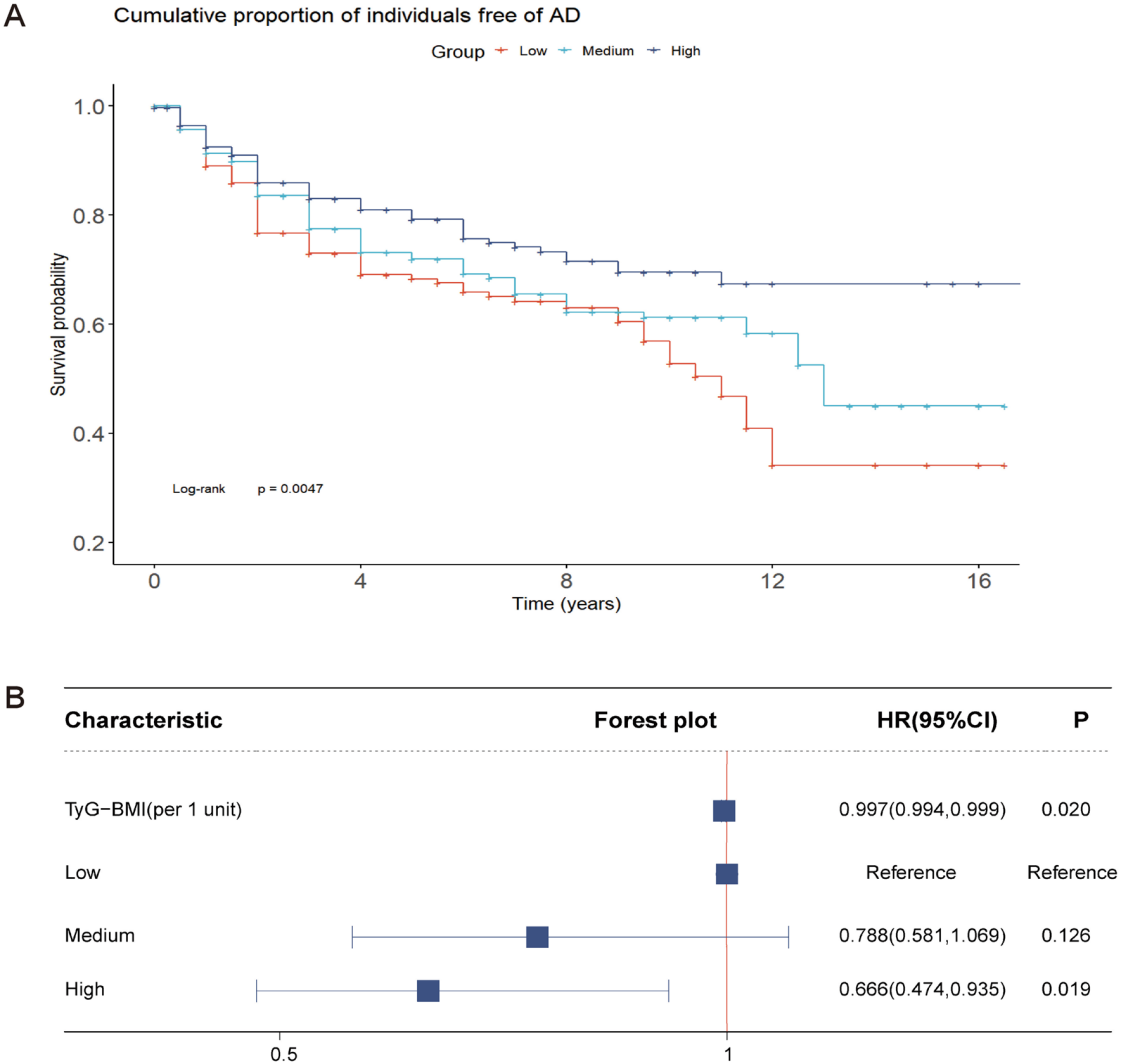
Association of TyG-BMI index with survival rates and risk of AD. (**A**) Survival probability of Alzheimer’s Disease over time across different TyG-BMI index levels. (**B**) The cumulative incidence of Alzheimer’s Disease based on Cox regression of TyG-BMI index.

During a follow-up period of up to 16 years, we analyzed patient groups with different TyG-BMI levels using the Kaplan–Meier method. The results showed that the group of patients with high TyG-BMI levels exhibited higher survival rates during the follow-up period, and the survival curves showed longer survival times compared to the group of patients with low TyG-BMI levels (Fig. 3A).

Adjusted Cox regression was employed to compare the likelihood of developing AD across different TyG-BMI index levels. Findings revealed that in the adjusted model, when considering the TyG-BMI index as a continuous variable, each 1-unit increase in the index correlated with a 0.3% decrease in incident AD risk (HR 0.996 [0.994, 0.999]). Utilizing the TyG-BMI index as a categorical variable, with low group as the baseline, a greater reduction in AD risk was observed in high group (HR: 0.625 [0.444, 0.878]) (Fig. 3B). Additionally, among individuals older than 60 years old, not carrying the *APOE ε4* gene, no diabetes or mildly cognitively impaired, the risk of developing AD decreased with each unit increase in the TyG-BMI index. However, these associations were marginally significant (Supplementary file Table 8).

### Causal mediation analyses

**Figure 4:**
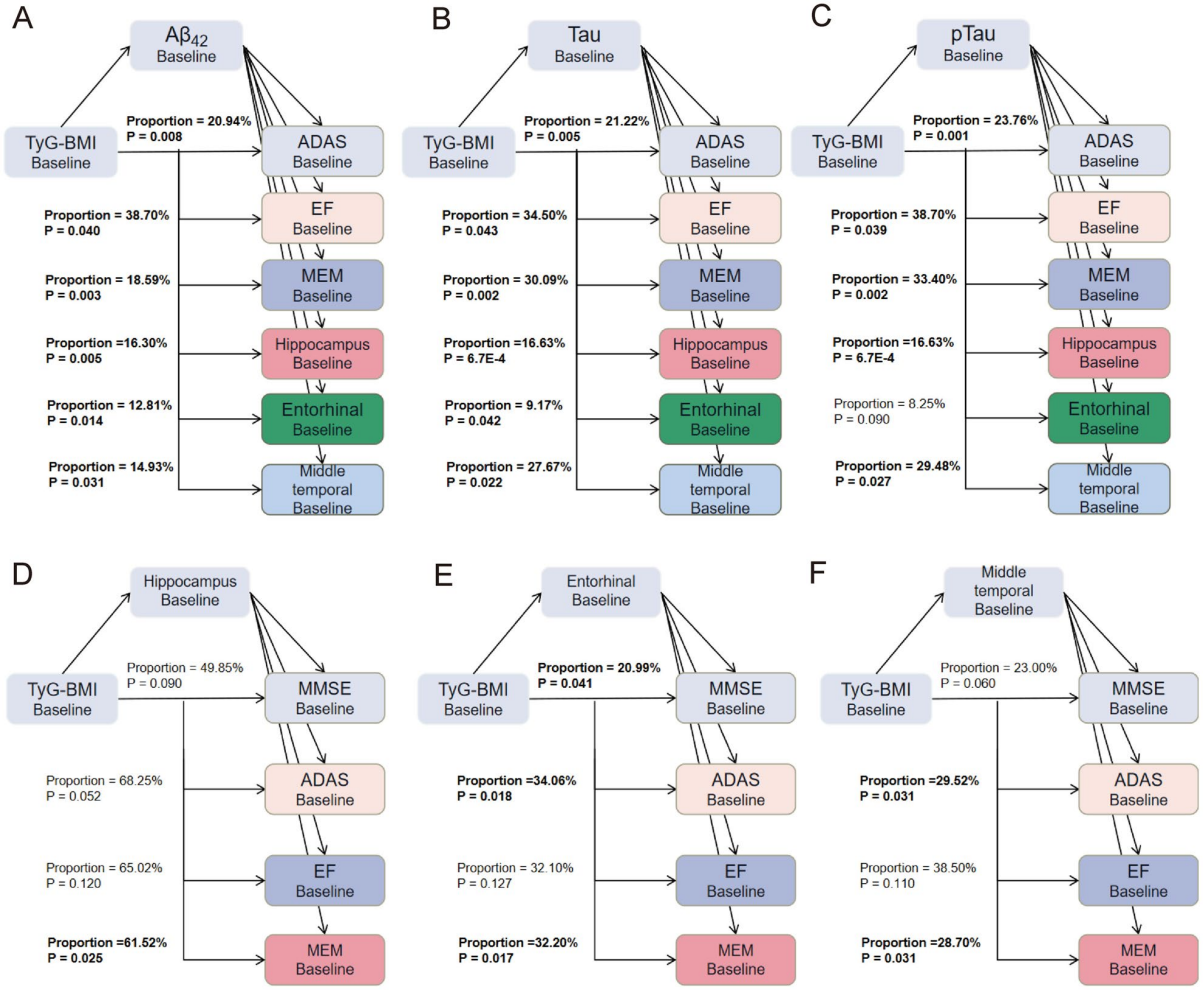
Mediation analyses of TyG-BMI index and cognition/brain structure. (**A**–**C**) Mediation analyses of TyG-BMI index and cognition as well as brain structure with biomarkers as mediators. (**D**–**F**) Mediation analyses of TyG-BMI index and cognition with brain structure as mediators. TyG-BMI index, triglyceride glucose-body mass index; Aβ42, Amyloid-42; pTau, phosphorylated-tau; Tau, total-tau; MMSE, mini-mental state examination; ADAS, Alzheimer’s Disease Assessment Scale-Cognitive Subscale Question; MEM, memory function.

We explored whether the association between TyG-BMI index and cognition as well as brain structure is mediated by AD pathology, and whether the correlation between TyG-BMI index and cognition is mediated by brain structure. Analyses showed that Aβ_42_, Tau, pTau, and Tau/pTau all mediated changes in cognition and brain structure (Fig. 4A–C, Supplementary file Table 9). Tau proteins as well as pTau proteins have a greater impact on cognition compared to Aβ_42_. We found that the TyG-BMI index affects the hippocampus and the entorhinal cortex through Tau/Aβ_42_ and pTau/Aβ_42_. Specifically, the proportion of the total effect on the hippocampus mediated by Tau/Aβ_42_ is 32.60%, and by pTau/Aβ_42_ is 29.70%. The proportion of the total effect on the entorhinal cortex mediated by Tau/Aβ_42_ is 24.82%, and by pTau/Aβ_42_ is 21.66%. (Supplementary file Table 9). We also found that the middle temporal mediated the association between TyG-BMI index and ADAS (Proportion = 29.52%) and MEM (Proportion = 28.70%) (Fig. 4D–F, Supplementary file Table 10).

## Discussion

In this study, we investigated the association between the TyG-BMI index and AD pathology, cognition, and brain structure. Our findings indicate that higher TyG-BMI levels are significantly associated with lower levels of Tau and pTau, as well as higher levels of Aβ_42_. These findings suggest that the TyG-BMI index may reflect systemic metabolic disturbances, such as IR and obesity, which are known to influence AD pathology. We observed that higher TyG-BMI index levels were significantly associated with a slower rate of cognitive decline as well as slower atrophy in the entorhinal cortex and middle temporal volume. Additionally, Aβ_42_, Tau, and pTau significantly mediated the total effect of the TyG-BMI index on cognition and brain structure. These AD pathological proteins may play a crucial role in linking metabolic health to neurodegeneration and cognitive decline.

The association of higher TyG-BMI index levels with lower Tau and pTau levels, as well as higher Aβ_42_, indicates a potentially protective metabolic effect on AD pathology. Our results are consistent with findings from a cohort study in China, which found a positive association between TyG index and Aβ_42_, as well as a positive correlation with the Aβ_42_/Aβ_40_ ratio, which better reflects the metabolism of β-amyloid^11^. IR has been found to promote the deposition of Aβ by decreasing Aβ clearance and increasing its oligomerization propensity^25,26^. Additionally, IR promotes the formation of Aβ fibrils by inducing GM1 ganglioside clustering in presynaptic membranes^26^. This creates a feedback loop where Aβ oligomers exacerbate brain IR, leading to progressive Aβ deposition^27^. Moreover, IR promotes Tau hyperphosphorylation through the activity of kinases such as GSK-3β, further establishing the link between IR and Tau pathology^28,29^. These evidences collectively suggest that metabolic disturbances reflected by the TyG-BMI index may influence AD pathology through mechanisms involving both Aβ and Tau.

We also found that the higher TyG-BMI levels were linked to slower atrophy in the entorhinal cortex and middle temporal volumes, which are critical regions affected in AD. Several investigations have explored the association between IR and brain structure of AD^30,31^. Studies have shown that IR is associated with cognitive dysfunction and brain atrophy, particularly in the hippocampus and temporal lobe regions, which are crucial for memory and cognitive functions^32^.

In terms of cognitive measures, we found that higher TyG-BMI index levels were associated with slower cognitive decline. This contrasts with several studies that have reported higher TyG levels are associated with increased cognitive impairment and AD risk^33,34^. For instance, a meta-analysis indicated that higher TyG levels are significantly associated with a higher risk of cognitive impairment and dementia^35^. One possible explanation for these contrasting findings is the role of BMI in modulating the effects of IR. While high BMI is generally considered a risk factor for metabolic syndrome, it can also reflect higher muscle mass and better overall nutritional status, which may confer some neuroprotective effects. Research has shown that a higher BMI in older adults can be associated with a protective effect against cognitive decline. For example, a study on Parkinson’s disease patients found that those with higher BMI at diagnosis experienced slower cognitive decline and had a lower risk of developing dementia compared to those with lower BMI^36^. Additionally, mild IR may trigger compensatory mechanisms that protect brain function in early stages, while severe IR leads to harmful effects^37^.

In the interaction analysis, we found significant interactions between age and TyG-BMI index with Aβ_42_. This interaction indicates that the influence of TyG-BMI on Aβ_42_ levels is more pronounced in older adults. This is supported by findings from previous studies showing that metabolic factors such as IR and BMI have different impacts on AD pathology across age groups. The older adults with better metabolic health may experience a slower accumulation of AD-related pathologies^38,39^. Similarly, the interaction between cognitive diagnosis and TyG-BMI with Tau suggests that individuals with MCI exhibit a different pattern of Tau accumulation in relation to their TyG-BMI levels compared to cognitively normal individuals. This suggests the need to consider cognitive status when assessing metabolic effects on AD pathology. Previous research has shown that individuals with MCI often have varying degrees of metabolic dysfunction, which can differentially affect the progression of tau pathology^40^.

Additionally, Aβ_42_, Tau, and pTau significantly mediated the association of the TyG-BMI index on cognition and brain structure. The TyG-BMI index can mediate cognitive changes by influencing brain structure. We found that the middle temporal lobe mediated the association between TyG-BMI index and ADAS (29.52%) and MEM (28.70%). The middle temporal plays an important role in memory and cognitive function, with increased volume positively associated with MEM scores and negatively associated with ADAS scores. Although studies have shown that IR can affect Aβ deposition and Tau phosphorylation^13^, leading to neurodegenerative processes^38,39^. However, the effects of IR vary from person to person. In some cases, IR may induce a range of compensatory mechanisms, such as increasing insulin levels in the brain, which in turn may have a protective effect on neurons^32,41^.

The association of FBG and TG levels with AD should not be overlooked in the TyG-BMI index as an indicator calculated from them. In a prolonged follow-up of ASPirin in Reducing Events in the Elderly and the UK Biobank cohorts, older individuals exhibiting elevated TG levels experienced a reduced risk of dementia and decelerated cognitive decline^42^. Besides, the Rotterdam Study revealed that elevated glucose levels were linked to increased IR burden and the risk of AD. However, this association was observed mainly in short-term follow-up results (< 3 years), while in follow-ups exceeding 5.5 years, elevated blood glucose levels and increased IR burden were reduced by 39% and 30%, respectively, suggesting possible time-dependence^43^.

IR occurs when the cellular response to insulin is weakened, resulting in blood glucose not entering the cell efficiently, which in turn increases insulin levels. Insulin’s protective effect on the brain could explain our results^44^. Insulin promotes the clearance and degradation of Aβ proteins by activating insulin receptors in glial cells^45,46^. Besides, IR induces neuronal damage and apoptosis, exacerbating Aβ protein release^47^. Damaged neurons release more Aβ protein, accelerating Aβ protein aggregation and plaque formation^48^. Additionally, insulin regulates the transcription and translation of Tau proteins through the PI3K/Akt signaling pathway^49^. IR interferes with this pathway’s normal function, resulting in abnormal expression and phosphorylation of Tau proteins^50^. Moreover, IR-induced oxidative stress leads to the accumulation of free radicals and reactive oxidants, causing DNA, lipid, and protein damage in brain cells^51,52^. This oxidative damage not only induces neuronal degeneration and apoptosis but also accelerates the aggregation of Aβ proteins and the phosphorylation of Tau proteins^53^.

There are several limitations to this study. First, because this study was a single-center study, the results still need to be validated in other larger longitudinal cohorts to ensure generalizability. Second, due to database limitations, we were not able to compare TyG-BMI index with other current IR measurement techniques. Third, we did not consider the effects of cerebrovascular disease, lifestyle, dietary habits, and physical activity. Additionally, the statistical methods used, including MLR, Cox models, and mediation analysis, have inherent limitations. Unmeasured confounders may affect results, and interpretations should be cautious as they suggest potential pathways rather than definitive causality. Mediation analysis, in particular, may have limitations due to model assumptions. These preliminary findings require validation in larger, diverse cohorts and experimental studies.

## Conclusions

Based on the presented findings, the TyG-BMI index is associated with AD pathology, cognition and brain structures such as the hippocampus. In addition, the TyG-BMI index may modulate cognitive and structural brain changes by impacting pathologic proteins. These results propose that monitoring the TyG-BMI index could serve as a valuable indicator for assessing AD risk and guiding timely interventions for metabolic disorders, thereby aiding in AD prevention.

### Supplementary Information

Supplementary Tables.

## Data Availability

Raw data supporting the obtained results are available at the corresponding author.
