# Supplementary material for: Association of triglyceride glucose-body mass index with Alzheimer’s disease pathology, cognition and brain structure in non-demented people
Source: Sci Rep. 2024 Jul 12;14:16097. doi: 10.1038/s41598-024-67052-3 (PMC11245502; doi:10.1038/s41598-024-67052-3)
Supplement: Supplementary file 1 — Supplementary Tables. [file 41598_2024_67052_MOESM1_ESM.docx]

Table 1 Association between TyG-BMI index and AD pathology, cognition and brain structure

| Variance | Model1 | | Model2 | |
| --- | --- | --- | --- | --- |
|  | β | P | β | P |
| **AD pathology** |  |  |  |  |
| Aβ_42_ | 0.121 | 3.87E-04 | 0.096 | 0.003 |
| Tau | -0.143 | 2.85E-5 | -0.134 | 6.38E-05 |
| pTau | -0.148 | 1.38E-5 | -0.148 | 2.62E-05 |
| Tau/Aβ_42_ | -0.161 | 2.11E-6 | -0.144 | 5.38E-06 |
| pTau/Aβ_42_ | -0.165 | 1.25E-6 | -0.145 | 3.84E-06 |
| pTau/Tau | -0.135 | 7.29E-5 | -0.127 | 1.42E-04 |
| **Cognition** |  |  |  |  |
| MMSE | 0.038 | 0.267 | 0.071 | 0.030 |
| ADAS | 0.071 | 0.038 | -0.093 | 0.002 |
| EF | 0.018 | 0.591 | 0.068 | 0.042 |
| MEM | 0.065 | 0.056 | 0.095 | 0.001 |
| **Brain structure** |  |  |  |  |
| Hippocampus | 0.132 | 3.04E-4 | 0.129 | 3.17E-04 |
| Middle temoprary | 0.068 | 0.065 | 0.077 | 0.022 |
| Entorhinal | 0.101 | 0.006 | 0.098 | 0.006 |

Abbreviations: TyG-BMI index, triglyceride glucose-body mass index; AD, Alzheimer’s Disease; Aβ_42_, Amyloid-42; pTau, phosphorylated-tau; Tau, total-tau; MMSE, mini-mental state examination, ADAS, Alzheimer’s Disease Assessment Scale; EF, executive function; MEM, memory function.

Model 1 unadjusted.

Model 2 adjusted for sex, age, ethnicity, Apolipoprotein E4, cognitive dignosis, diabetes, education, smoking ,drinking, hypertension and cardiovascular disease, brain structure additionally adjusted for intracerebral volume.

Table 2 Interaction effects table for TyG-BMI with pathology, cognition, and brain structure

| Variance | Age | Sex | Dignosis | APOE ε4 | Ethnicity | Cardiovascu-lar | Diabetes | Alcohol | Smoking |
| --- | --- | --- | --- | --- | --- | --- | --- | --- | --- |
|  | P for interaction | P for interaction | P for interaction | P for interaction | P for interaction | P for interaction | P for interaction | P for interaction | P for interaction |
| **AD pathology** |  |  |  |  |  |  |  |  |  |
| Aβ_42_ | 0.018 | 0.536 | 0.182 | 0.065 | 0.463 | 0.537 | 0.081 | 0.754 | 0.300 |
| Tau | 0.919 | 0.498 | 0.042 | 0.839 | 0.249 | 0.491 | 0.415 | 0.79 | 0.903 |
| pTau | 0.816 | 0.346 | 0.051 | 0.782 | 0.345 | 0.532 | 0.342 | 0.659 | 0.990 |
| Tau/Aβ_42_ | 0.215 | 0.353 | 0.297 | 0.448 | 0.786 | 0.836 | 0.875 | 0.432 | 0.730 |
| pTau/Aβ_42_ | 0.209 | 0.426 | 0.332 | 0.383 | 0.745 | 0.785 | 0.709 | 0.444 | 0.576 |
| pTau/Tau | 0.157 | 0.057 | 0.255 | 0.824 | 0.596 | 0.329 | 0.590 | 0.491 | 0.964 |
| **Cognition** |  |  |  |  |  |  |  |  |  |
| MMSE | 0.485 | 0.356 | 0.49 | 0.081 | 0.233 | 0.750 | 0.943 | 0.687 | 0.782 |
| ADAS | 0.072 | 0.931 | 0.279 | 0.546 | 0.327 | 0.533 | 0.772 | 0.164 | 0.433 |
| EF | 0.515 | 0.905 | 0.933 | 0.769 | 0.766 | 0.525 | 0.159 | 0.918 | 0.514 |
| MEM | 0.654 | 0.889 | 0.132 | 0.656 | 0.841 | 0.900 | 0.703 | 0.223 | 0.852 |
| **Brain structure** |  |  |  |  |  |  |  |  |  |
| Hippocampus | 0.406 | 0.975 | 0.827 | 0.092 | 0.611 | 0.911 | 0.993 | 0.875 | 0.886 |
| Middle temoprary | 0.126 | 0.257 | 0.509 | 0.401 | 0.955 | 0.456 | 0.981 | 0.682 | 0.926 |
| Entorhinal | 0.626 | 0.958 | 0.343 | 0.482 | 0.688 | 0.589 | 0.983 | 0.919 | 0.987 |

Abbreviations: TyG-BMI index, triglyceride glucose-body mass index; AD, Alzheimer’s Disease; APOE ε4, Apolipoprotein E; Aβ_42_, Amyloid-42; pTau, phosphorylated-tau; Tau, total-tau; MMSE, mini-mental state examination, ADAS, Alzheimer’s Disease Assessment Scale; EF, executive function; MEM, memory function.

Table 3 Subgroup analysis of association between TyG-BMI index and AD pathology.

| Characteristic | | Aβ_42_ | | Tau | | pTau | | pTau/Aβ_42_ | | Tau/Aβ_42_ | | pTau/Tau | |
| --- | --- | --- | --- | --- | --- | --- | --- | --- | --- | --- | --- | --- | --- |
|  |  | β | P | β | P | β | P | β | P | β | P | β | P |
| Age | <60 | 0.552 | **0.017** | -0.083 | 0.698 | -0.142 | 0.501 | -0.376 | 0.097 | -0.379 | 0.104 | -0.382 | 0.067 |
|  | ≥60 | 0.076 | **0.021** | -0.136 | **7.86E-5** | -0.139 | **4.32E-5** | -0.133 | **3.51E-5** | -0.133 | **2.74E-5** | -0.115 | **7.17E-4** |
| Sex | Male | 0.115 | **0.008** | -0.178 | **8.29E-5** | -0.192 | **1.99E-5** | -0.188 | **2.62E-5** | -0.175 | **3.67E-5** | -0.188 | **2.62E-5** |
|  | Female | 0.064 | 0.199 | -0.083 | 0.107 | -0.082 | 0.107 | -0.099 | **0.038** | -0.104 | **0.028** | -0.062 | 0.232 |
| Dignosis | CN | 0.136 | **0.024** | -0.063 | 0.315 | -0.082 | 0.193 | -0.168 | **0.005** | -0.174 | **0.003** | -0.088 | 0.149 |
|  | MCI | 0.088 | **0.027** | -0.165 | **5.63E-5** | -0.169 | **3.38E-5** | -0.155 | **7.53E-5** | -0.154 | **7.72E-5** | -0.154 | **1.77E-5** |
| APOE ε4 | APOE ε4(+) | 0.189 | **3.28E-4** | -0.125 | **0.018** | -0.130 | **0.014** | -0.140 | **0.008** | -0.144 | **0.006** | -0.123 | **0.024** |
|  | APOE ε4(-) | 0.052 | 0.263 | -0.146 | **0.002** | -0.155 | **0.001** | -0.173 | **1.88E-5** | -0.192 | **2.10E-5** | -0.132 | **0.005** |
| Diabetes | No | 0.123 | **2.96E-4** | -0.122 | **5.49E-4** | -0.126 | **3.17E-4** | -0.146 | **1.084-5** | -0.150 | **5.11E-6** | -0.117 | **9.64E-4** |
|  | Yes | -0.059 | 0.530 | -0.167 | 0.093 | -0.177 | 0.068 | -0.107 | 0.215 | -0.093 | 0.285 | -0.170 | 0.078 |

Abbreviations: TyG-BMI index, triglyceride glucose-body mass index; AD, Alzheimer’s Disease; APOE ε4, Apolipoprotein E; CN, cognitive normal; MCI, mild cognitive impartment; Aβ_42_, Amyloid-42; pTau, phosphorylated-tau; Tau, total-tau.

All factors adjusted for sex, age, ethnicity, Apolipoprotein E4, cognitive dignosis, diabetes, education, smoking ,drinking, hypertension and cardiovascular disease except itself.

Table 4 Subgroup analysis of association between TyG-BMI index and cognition.

| Characteristic | | MMSE | | ADAS | | EF | | MEM | |
| --- | --- | --- | --- | --- | --- | --- | --- | --- | --- |
|  |  | β | P | β | P | β | P | β | P |
| Age | <60 | 0.139 | 0.574 | -0.288 | 0.199 | -0.051 | 0.796 | 0.052 | 0.796 |
|  | ≥60 | 0.068 | **0.041** | -0.077 | **0.019** | 0.062 | 0.069 | 0.091 | **0.003** |
| Sex | Male | 0.099 | **0.023** | -0.081 | 0.066 | 0.057 | 0.202 | 0.094 | **0.021** |
|  | Female | 0.063 | 0.209 | -0.114 | **0.022** | 0.087 | **0.091** | 0.112 | **0.015** |
| Dignosis | CN | -0.007 | 0.901 | -0.078 | 0.198 | 0.083 | 0.174 | 0.062 | 0.256 |
|  | MCI | 0.102 | **0.018** | -0.111 | **0.009** | 0.071 | 0.093 | 0.128 | **0.002** |
| APOE ε4 | APOE ε4(+) | -0.007 | 0.890 | -0.079 | 0.115 | 0.047 | 0.370 | 0.076 | 0.097 |
|  | APOE ε4(-) | 0.131 | **0.003** | -0.103 | **0.018** | 0.079 | 0.071 | 0.103 | **0.010** |
| Diabetes | No | 0.068 | **0.049** | -0.083 | **0.014** | 0.087 | **0.013** | 0.095 | **0.002** |
|  | Yes | 0.077 | 0.437 | -0.116 | 0.234 | -0.063 | 0.540 | 0.039 | 0.684 |

Abbreviations: TyG-BMI index, triglyceride glucose-body mass index; AD, Alzheimer’s Disease; APOE ε4, Apolipoprotein E; CN, cognitive normal; MCI, mild cognitive impartment; MMSE, mini-mental state examination, ADAS, Alzheimer’s Disease Assessment Scale; EF, executive function; MEM, memory function.

All factors adjusted for sex, age, ethnicity, Apolipoprotein E4, cognitive dignosis, diabetes, education, smoking ,drinking, hypertension and cardiovascular disease except itself.

Table 5 Subgroup analysis of association between TyG-BMI index and brain structure.

| Characteristic | | Hippocampus | | Entorhinal | | Middle temopral | |
| --- | --- | --- | --- | --- | --- | --- | --- |
|  |  | β | P | β | P | β | P |
| Age | <60 | 0.212 | 0.428 | -0.035 | 0.882 | 0.219 | 0.303 |
|  | ≥60 | 0.119 | **0.001** | 0.103 | **0.005** | 0.068 | 0.074 |
| Sex | Male | 0.120 | **0.001** | 0.087 | 0.076 | 0.034 | 0.483 |
|  | Female | 0.146 | **0.007** | 0.110 | **0.051** | 0.135 | **0.011** |
| Dignosis | CN | 0.147 | **0.017** | 0.194 | **0.001** | 0.061 | 0.240 |
|  | MCI | 0.133 | **0.003** | 0.068 | 0.132 | 0.081 | 0.057 |
| APOE ε4 | APOE ε4(+) | 0.170 | **0.002** | 0.098 | 0.080 | 0.017 | 0.734 |
|  | APOE ε4(-) | 0.102 | **0.033** | 0.103 | **0.031** | 0.100 | **0.026** |
| Diabetes | No | 0.124 | **9.55E-4** | 0.093 | **0.014** | 0.076 | **0.030** |
|  | Yes | 0.209 | 0.053 | 0.119 | 0.294 | 0.069 | 0.506 |

Abbreviations: TyG-BMI index, triglyceride glucose-body mass index; AD, Alzheimer’s Disease; APOE ε4, Apolipoprotein E; CN, cognitive normal; MCI, mild cognitive impartment.

All factors adjusted for sex, age, ethnicity, Apolipoprotein E4, cognitive dignosis, diabetes, education, smoking ,drinking, hypertension, cardiovascular disease and intracerebral volume except itself.

Table 6 Longitudinal relationship between TyG-BMI index with AD pathology, cognitive measures and brain structure.

|  | β | P |
| --- | --- | --- |
| **AD pathology** |  |  |
| Aβ_42_ | 0.001 | 0.873 |
| Tau | 0.003 | 0.515 |
| pTau | 0.006 | 0.232 |
| Tau/Aβ_42_ | -0.004 | 0.537 |
| pTau/Aβ_42_ | -0.007 | 0.325 |
| pTau/Tau | 0.011 | 0.199 |
| **Cognition** |  |  |
| MMSE | 0.045 | 6.36E-5 |
| ADAS | -0.046 | 5.88E-6 |
| MEM | 0.015 | 0.011 |
| EF | 0.024 | 3.36E-5 |
| **Brain structure** |  |  |
| Hippocampus | 0.006 | 0.058 |
| Middle temopral | 0.013 | 0.036 |
| Entorhinal | 0.014 | 0.007 |

Abbreviations: TyG-BMI index, triglyceride glucose-body mass index; AD, Alzheimer’s Disease; Aβ42, Amyloid-42; pTau, phosphorylated-tau; Tau, total-tau; MMSE, mini-mental state examination, ADAS, Alzheimer’s Disease Assessment Scale; EF, executive function; MEM, memory function.

All factors adjusted for sex, age, ethnicity, Apolipoprotein E4, cognitive dignosis, diabetes, education, smoking ,drinking, hypertension and cardiovascular disease, brain structure additionally adjusted for intracerebral volume.

Table 7 Number of AD biomarkers, cognition, and brain imaging participants at different follow-up times.

|  | Baseline | 1 year | 2 year | 3 year | 4 year | 5 year | 6 year | 7 year | 8 year | 9 year | 10 year | 11 year | 12 year | 13 year | 14 year | 15 year | 16 year |
| --- | --- | --- | --- | --- | --- | --- | --- | --- | --- | --- | --- | --- | --- | --- | --- | --- | --- |
| **AD pathology** |  |  |  |  |  |  |  |  |  |  |  |  |  |  |  |  |  |
| Aβ_42_ | 404 | 195 | 261 | 57 | 124 | 30 | 18 | 11 | 5 | 9 | 4 | - | - | - | - | - | - |
| Tau | 504 | 217 | 337 | 63 | 161 | 34 | 21 | 15 | 7 | 12 | 4 | - | - | - | - | - | - |
| pTau | 503 | 217 | 336 | 63 | 160 | 34 | 21 | 15 | 7 | 12 | 4 | - | - | - | - | - | - |
| Tau/Aβ_42_ | 403 | 163 | 265 | 57 | 124 | 30 | 18 | 11 | 5 | 9 | 4 | - | - | - | - | - | - |
| pTau/Aβ_42_ | 403 | 163 | 265 | 57 | 124 | 30 | 18 | 11 | 5 | 9 | 4 | - | - | - | - | - | - |
| pTau/Tau | 403 | 163 | 265 | 57 | 124 | 30 | 18 | 11 | 5 | 9 | 4 | - | - | - | - | - | - |
| **Cognition** |  |  |  |  |  |  |  |  |  |  |  |  |  |  |  |  |  |
| MMSE | 855 | 656 | 540 | 390 | 300 | 130 | 80 | 62 | 52 | 27 | 27 | 6 | 4 | 5 | 3 | 2 | 2 |
| ADAS | 855 | 654 | 540 | 388 | 298 | 130 | 80 | 60 | 52 | 26 | 27 | 6 | 4 | 5 | 3 | 2 | 2 |
| MEM | 855 | 802 | 710 | 508 | 441 | 214 | 190 | 139 | 116 | 71 | 64 | 35 | 14 | 14 | 2 | 8 | 3 |
| EF | 855 | 802 | 710 | 508 | 441 | 214 | 190 | 139 | 116 | 71 | 64 | 35 | 14 | 14 | 2 | 8 | 3 |
| **Brain structure** |  |  |  |  |  |  |  |  |  |  |  |  |  |  |  |  |  |
| Hippocampus | 729 | 555 | 456 | 169 | 210 | 55 | 52 | 24 | 17 | 12 | 7 | 6 | 3 | 4 | 2 | 1 | - |
| Entorhinal | 718 | 530 | 387 | 155 | 172 | 50 | 53 | 22 | 13 | 11 | 4 | 6 | 3 | 4 | 2 | 1 |  |
| Middle Temporal | 718 | 530 | 387 | 155 | 172 | 50 | 53 | 22 | 13 | 11 | 4 | 6 | 3 | 4 | 2 | 1 | - |

Abbreviations: TyG-BMI index, triglyceride glucose-body mass index; AD, Alzheimer’s Disease; Aβ_42_, Amyloid-42; pTau, phosphorylated-tau; Tau, total-tau; MMSE, mini-mental state examination, ADAS11, Alzheimer’s Disease Assessment Scale; ; EF, executive function; MEM, memory function.

Table 8 The cumulative incidence of Alzheimer's Disease based on Cox regression of TyG-BMI index

| Characteristic | HR(95%CI) | P |
| --- | --- | --- |
| TyG-BMI(per 1 unit) | 0.996(0.994,0.999) | 0.019 |
| Low | Reference | Reference |
| Medium | 0.793(0.584,1.076) | 0.136 |
| High | 0.625(0.444,0.878) | 0.017 |
| Age |  |  |
| <60 | 0.980(0.957,1.002) | 0.080 |
| ≥60 | 0.997(0.994,1.000) | 0.050 |
| Sex |  |  |
| Male | 0.996(0.992,1.000) | 0..082 |
| Female | 0.997(0.993,1.002) | 0.219 |
| Dignosis |  |  |
| Cognitive normal | 0.994(0.983,1.005) | 0.292 |
| Mild cognitive impartment | 0.997(0.994,1.000) | 0.042 |
| APOE ε4 |  |  |
| APOE ε4(-) | 0.994(0.988,0.999) | 0.029 |
| APOE ε4(+) | 00.998(0.995,1.002) | 0.304 |
| Diabetes |  |  |
| No | 0.995(0.992,0.999) | 0.005 |
| Yes | 0.998(0.990,1.005) | 0.568 |

Abbreviations: TyG-BMI index, triglyceride glucose-body mass index; AD, Alzheimer’s Disease; APOE ε4, Apolipoprotein E

All factors adjusted for sex, age, ethnicity, Apolipoprotein E4, cognitive dignosis, diabetes, education, smoking ,drinking, hypertension and cardiovascular disease except itself.Table 9 Mediation analyses of TyG-BMI index and cognitive measurements as well as brain structure with AD biomarkers as mediators in non-dementia participants.

|  |  | a | P | b | P | c | P | c' | P | Proportion(%) | P |
| --- | --- | --- | --- | --- | --- | --- | --- | --- | --- | --- | --- |
| Aβ_42_ | MMSE | 1.254 | 0.003 | 3.70E-4 | 2.38E-4 | 0.003 | 0.030 | 0.002 | 0.070 | 16.63% | 0.036 |
|  | ADAS | 1.254 | 0.003 | -0.002 | 2.64E-10 | -0.009 | 0.004 | -0.007 | 0.021 | 20.94% | 0.008 |
|  | EF | 1.254 | 0.003 | 4.52E-4 | 4.78E-16 | 0.001 | 0.042 | 8.09E-4 | 0.202 | 38.70% | 0.040 |
|  | MEM | 1.254 | 0.003 | 2.49E-4 | 2.49E-10 | 0.002 | 0.001 | 0.001 | 0.008 | 18.59% | 0.003 |
|  | Hippocampus | 1.347 | 0.004 | 0.403 | 3.47E-9 | 3.165 | 3.02E-4 | 2.652 | 0.002 | 16.30% | 0.005 |
|  | Middle temopral | 1.253 | 0.006 | 0.657 | 1.64E-4 | 4.933 | 0.023 | 4.152 | 0.054 | 14.93% | 0.031 |
|  | Entorhinal | 1.253 | 0.006 | 0.174 | 1.10E-4 | 1.540 | 0.006 | 1.336 | 0.016 | 12.81% | 0.014 |
| Tau | MMSE | -0.349 | 6.38E-5 | -0.002 | 4.53E-6 | 0.003 | 0.030 | 0.002 | 0.116 | 26.755 | 0.037 |
|  | ADAS | -0.349 | 6.38E-5 | 0.006 | 1.51E-6 | -0.009 | 0.004 | -0.007 | 0.023 | 21.22% | 0.005 |
|  | EF | -0.349 | 6.38E-5 | -0.001 | 2.62E-8 | 0.001 | 0.042 | 8.53E-4 | 0.189 | 34.50% | 0.043 |
|  | MEM | -0.349 | 6.38E-5 | -0.001 | 1.51E-14 | 0.002 | 0.001 | 0.001 | 0.022 | 30.09% | 0.002 |
|  | Hippocampus | -0.368 | 1.01E-4 | -1.600 | 2.39E-6 | 3.165 | 3.02E-4 | 2.630 | 0.003 | 16.63% | 6.70E-4 |
|  | Middle temopral | -0.324 | 5.53E-4 | -4.484 | 1.14E-7 | 4.993 | 0.023 | 3.536 | 0.099 | 27.67% | 0.022 |
|  | Entorhinal | -0.324 | 5.53E-4 | -0.519 | 0.018 | 1.540 | 0.006 | 1.394 | 0.013 | 9.17% | 0.042 |
| pTau | MMSE | -0.041 | 2.62E-5 | -0.020 | 3.08E-6 | 0.003 | 0.030 | 0.002 | 0.126 | 29.46% | 0.025 |
|  | ADAS | -0.041 | 2.62E-5 | 0.059 | 1.90E-07 | -0.009 | 0.004 | -0.007 | 0.029 | 23.76% | 0.001 |
|  | EF | -0.041 | 2.62E-5 | -0.013 | 5.44E-9 | 0.001 | 0.042 | 8.04E-4 | 0.216 | 38.70% | 0.039 |
|  | MEM | -0.041 | 2.62E-5 | -0.013 | 2.44E-15 | 0.002 | 0.001 | 0.001 | 0.028 | 33.40% | 0.002 |
|  | Hippocampus | -0.044 | 2.72E-5 | -13.719 | 8.27-6 | 3.165 | 3.02E-4 | 2.627 | 0.003 | 16.30% | 2.00E-16 |
|  | Middle temopral | -0.039 | 2.00E-4 | -39.773 | 1.92E-7 | 4.993 | 0.023 | 3.460 | 0.107 | 29.48% | 0.027 |
|  | Entorhinal | -0.039 | 2.00E-4 | -4.147 | 0.037 | 1.540 | 0.006 | 1.406 | 0.012 | 8.25% | 0.090 |
| pTau/Aβ_42_ | MMSE | -8.70E-5 | 5.38E-6 | -13.148 | 2.35E-9 | 0.003 | 0.030 | 0.002 | 0.199 | 39.83% | 0.025 |
|  | ADAS | -8.70E-5 | 5.38E-6 | 42.590 | 5.30E-14 | -0.009 | 0.004 | -0.006 | 0.072 | 38.76% | 0.005 |
|  | EF | -8.70E-5 | 5.38E-6 | -9.761 | 2.00E-16 | 0.001 | 0.042 | 4.97E-4 | 0.435 | 61.60% | 0.039 |
|  | MEM | -8.70E-5 | 5.38E-6 | -8.107 | 2.00E-16 | 0.002 | 0.001 | 0.001 | 0.057 | 43.60% | 0.002 |
|  | Hippocampus | -9.48E-5 | 6.96E-6 | -1.06E+4 | 1.63E-12 | 3.165 | 3.02E-4 | 2.217 | 0.010 | 29.70% | 2.00E-16 |
|  | Middle temopral | -8.510 | 3.80E-5 | -2.39E+4 | 4.01E-10 | 4.993 | 0.023 | 2.966 | 0.165 | 38.90% | 0.017 |
|  | Entorhinal | -8.51E-5 | 3.80E-5 | -4.27E+3 | 1.68E-5 | 1.540 | 0.006 | 1.204 | 0.031 | 21.66% | 0.007 |
| Tau/Aβ_42_ | MMSE | -0.001 | 3.84E-6 | -1.492 | 5.39E-10 | 0.003 | 0.030 | 0.002 | 0.217 | 42.77% | 0.036 |
|  | ADAS | -0.001 | 3.84E-6 | 4.870 | 3.09E-15 | -0.009 | 0.004 | -0.005 | 0.084 | 41.55% | 0.009 |
|  | EF | -0.001 | 3.84E-6 | -1.126 | 2.00E-16 | 0.001 | 0.042 | 4.34E-4 | 0.493 | 67.80% | 0.033 |
|  | MEM | -0.001 | 3.84E-6 | -0.921 | 2.00E-16 | 0.002 | 0.001 | 8.54E-4 | 0.068 | 45.10% | 0.003 |
|  | Hippocampus | -8.65E-4 | 8.24E-6 | -1.26E+3 | 1.08E-14 | 3.165 | 3.02E-4 | 2.131 | 0.013 | 32.60% | 4.00E-4 |
|  | Middle temopral | -7.81E-5 | 3.56E-5 | -2.78E+3 | 2.66E-11 | 4.993 | 0.023 | 2.826 | 0.184 | 41.50% | 0.017 |
|  | Entorhinal | -7.81E-4 | 3.56E-5 | -528.622 | 1.06E-6 | 1.540 | 0.006 | 1.154 | 0.038 | 24.82% | 0.012 |
| pTau/Tau | MMSE | -2.55E-5 | 1.42E-4 | -21.672 | 6.18E-4 | 0.003 | 0.030 | 0.002 | 0.081 | 18.76% | 0.032 |
|  | ADAS | -2.55E-5 | 1.42E-4 | 85.277 | 1.67E-7 | -0.009 | 0.004 | -0.007 | 0.024 | 22.06% | 0.005 |
|  | EF | -2.55E-5 | 1.42E-4 | -16.845 | 4.24E-7 | 0.001 | 0.042 | 9.19E-4 | 0.158 | 30.60% | 0.039 |
|  | MEM | -2.55E-5 | 1.42E-4 | -15.912 | 1.34E-10 | 0.002 | 0.001 | 0.001 | 0.013 | 24.61% | 6.70E-4 |
|  | Hippocampus | -2.96E-5 | 3.99E-5 | -9.77E+3 | 0.030 | 3.165 | 3.02E-4 | 2.944 | 8.73E-4 | 6.93% | 0.074 |
|  | Middle temopral | -2.54E-5 | 3.61E-4 | -3.33E+4 | 0.003 | 4.993 | 0.023 | 4.157 | 0.056 | 14.80% | 0.026 |
|  | Entorhinal | -2.54E-5 | 3.61E-4 | -1.89E+3 | 0.513 | 1.540 | 0.006 | 1.518 | 0.007 | 1.28% | 0.743 |

Abbreviations: TyG-BMI index, triglyceride glucose-body mass index; AD, Alzheimer’s Disease; Aβ_42_, Amyloid-42; pTau, phosphorylated-tau; Tau, total-tau; MMSE, mini-mental state examination, ADAS, Alzheimer’s Disease Assessment Scale; EF, executive function; MEM, memory function.

Table 10 Mediation analyses of TyG-BMI index and cognitive measurements with brain structure as mediators in non-dementia participants.

|  |  | a | P | b | P | c | P | c' | P | Proportion(%) | P |
| --- | --- | --- | --- | --- | --- | --- | --- | --- | --- | --- | --- |
| Hippocampus | MMSE | 3.165 | 3.02E-4 | 3.85E-4 | 2.98E-12 | 0.002 | 0.094 | 1.04E-3 | 0.427 | 49.85% | 0.090 |
|  | ADAS | 3.165 | 3.02E-4 | -0.002 | 2.00E-16 | -0.007 | 0.049 | -0.002 | 0.522 | 68.25% | 0.052 |
|  | EF | 3.165 | 3.02E-4 | 2.52E-04 | 2.00E-16 | 0.001 | 0.124 | 3.02E-04 | 0.658 | 65.02% | 0.120 |
|  | MEM | 3.165 | 3.02E-4 | 2.37E-04 | 2.00E-16 | 0.001 | 0.024 | 4.57E-04 | 0.353 | 61.52% | 0.025 |
| Entorhinal | MMSE | 1.540 | 0.006 | 3.88E-04 | 9.45E-06 | 0.003 | 0.044 | 2.09E-03 | 0.112 | 20.99% | 0.041 |
|  | ADAS | 1.540 | 0.006 | -0.002 | 2.00E-16 | -0.009 | 0.010 | -0.006 | 0.075 | 34.06% | 0.018 |
|  | EF | 1.540 | 0.006 | 2.68E-04 | 6.24E-09 | 0.001 | 0.107 | 7.29E-04 | 0.292 | 32.10% | 0.127 |
|  | MEM | 1.540 | 0.006 | 2.94E-04 | 2.00E-16 | 0.001 | 0.008 | 9.28E-04 | 0.06 | 32.20% | 0.017 |
| Middle temopral | MMSE | 4.933 | 0.023 | 1.33E-04 | 2.80E-09 | 0.003 | 0.044 | 0.002 | 0.12 | 23.00% | 0.060 |
|  | ADAS | 4.933 | 0.023 | -5.41E-04 | 2.00E-16 | -0.009 | 0.01 | -6.00E-03 | 0.054 | 29.52% | 0.031 |
|  | EF | 4.933 | 0.023 | 9.57E-05 | 4.07E-16 | 0.001 | 0.107 | 6.67E-04 | 0.323 | 38.50% | 0.110 |
|  | MEM | 4.933 | 0.023 | 8.18E-05 | 2.00E-16 | 0.001 | 0.008 | 9.75E-04 | 0.046 | 28.70% | 0.031 |

Abbreviations: TyG-BMI index, triglyceride glucose-body mass index; AD, Alzheimer’s Disease; MMSE, mini-mental state examination, ADAS, Alzheimer’s Disease Assessment Scale; EF, executive function; MEM, memory function.
